# PROTOCOL: Effects of preventive nutrition interventions among adolescents on health and nutritional status in low‐ and middle‐income countries: a systematic review

**DOI:** 10.1002/CL2.195

**Published:** 2018-10-21

**Authors:** Rehana A Salam, Jai K Das, Omar Irfan, Zulfiqar A Bhutta

## Background

### The problem, condition or issue

Malnutrition is one of the most common causes of morbidity and mortality among children and adolescents (UNICEF 2005) and along with poor diet, it is now considered to be the largest risk factor responsible for the global burden of diseases (Forouzanfar 2015). A survey conducted among adolescents aged 12–15 years from 57 low‐ middle‐ income countries (LMICs) between 2003 and 2013 suggested that the prevalence of stunting was 10.2% while thinness was 5.5% (Caleyachetti 2018). Micronutrient deficiencies account for a substantial global burden of diseases, with iron and vitamin A deficiency being among the 15 leading causes of global morbidity and mortality (WHO 2002). More than two billion people, including both children and adolescents, suffer from micronutrient deficiencies in the developing world (Stanger 2009). In 2014, iron deficiency anaemia was one of the three most common causes of disability‐adjusted life years (DALYs) lost among adolescents along with other micronutrient deficiencies accounting for over 2,500 DALYs per 100,000 adolescents (WHO 2014b; Akseer 2017).

Adolescence is a critical age group with key changes in health and its determinants later in life. Adequate nutrition is vital for transition from adolescence to healthy adults as the consequences of malnutrition among children and adolescents include delayed growth, impaired cognitive maturation, lower intellectual quotient (IQ), behavioural problems and increased risk of contracting communicable diseases (Mengistu 2013;Onyango 2013). There are many underlying determinants of undernutrition including poverty, food insecurity, poor sexual and reproductive health, violence, and many infectious and non‐infectious diseases (Patton 2016). The quality of available diets in LMICs is also a challenge as diet is fairly restricted and comprised largely of cereals or legumes with few animal products and a limited access to a variety of fruits and vegetables (Ladipo 2000). Poverty in these settings also leads to limited ability to purchase and consume sufficient amounts of key nutrients. Food insecurity in these settings has also been linked to poor diet quality and uncertainty in the food environment related to inability to access adequate food sources for the sustainability of healthy and active living (Akseer 2017). Food choices and preferences are also determinants of malnutrition since in some settings, despite adequate food access, dietary choices lead to nutritional deficiencies. Adolescents globally are consuming less than adequate amounts of fruits and vegetables and alarmingly high levels of sodium and sugar (Akseer 2017). These poor dietary habits and eating choices pose further threat to the growing bodies. The burden of malnutrition is further complicated for the women and girls in LMIC settings owing to the their status and power in society compared to their male counterparts (Jayachandran 2015). Micronutrient deficiency is often referred to as ‘hidden hunger’ and has a global health impact on adolescents because its manifestations are less visible and usually begins to show when the condition is severe and has already led to serious health consequences.

A number of nutrition‐specific interventions to address malnutrition have been advocated and these include nutrition education and counselling, micronutrient supplementation, food fortification and macronutrient supplementation.

### The intervention

The following interventions (alone or in combination) have been advocated to prevent nutrition deficiencies:

► Nutrition education and counselling
► Micronutrient supplementation and fortification
► Macronutrient supplementation

#### Nutrition education and counselling

Dietary habits of adolescents are influenced by various factors including food environments, food advertisements, mass media messages, peers and social eating culture (Riebl 2015; Stang 2017). Nutritional concerns among adolescents include poor dietary habits; low intake of fruits, vegetables, fibre and calcium‐rich foods; high intake of foods high in fat and sugar; unhealthy dieting; and erratic eating behaviours, such as meal skipping (Stang 2017).

Nutrition education and counselling is a widely used strategy to improve nutritional status and change nutrition related behaviours (Story 2002). The strategy focuses primarily on promoting a healthy diet by increasing the diversity and amount of foods consumed. Nutrition education can help young people attain the knowledge and skills they need to make healthful food choices and develop lifelong healthy eating patterns. Nutrition education and counselling for adolescents have been delivered through various platforms including schools, communities, peer‐based networks, computer and web based education (Kroeze 2006; Oenema 2001; Pérez‐Rodrigo 2001).

#### Micronutrient supplementation and fortification

Supplementation refers to the provision of individual or mixture of nutrients separately from the diet while adding nutrients to staple foods is termed as fortification. Micronutrients can be supplemented in form of injections, tablets, capsules, syrups/liquids or powders (Blasbalg 2011). Oral iron supplements, being the most common and inexpensive, have been established as frontline prevention and treatment for iron‐deficiency anaemia (Peyrin‐Biroulet 2015). Other micronutrient most commonly supplemented include calcium, vitamin D, vitamin A, iodine, zinc and multiple micronutrients (MMN) (Haider 2017; Hess 2009; Reid 2014; Zimmermann 2015; Zimmermann, 2007).

Food fortification is the process in which micronutrients are added to processed foods. In many stances, this approach has led to ameliorating micronutrient deficiencies in the population with reasonable cost making it a very efficient public health intervention. Fortification could be mass fortification (that is adding micronutrients to foods that are commonly consumed such as flour, salt, sugar and cooking oil) or point‐of‐use fortification (that involves adding single‐dose packets of vitamins and minerals in powder form that can be sprinkled onto any ready to eat food consumed at home, school, nurseries, refugee camps or any other place where possible) (WHO 2014b; Zlotkin 2005).

#### Macronutrient supplementation

Macronutrient interventions include supplementary feeding, balanced energy and protein supplementation and lipid based nutrition supplementation (LNS). Supplementary feeding is the provision of extra food to children or families beyond the normal ration of their home diets, and can take place in the home, feeding centres, healthcare centres and schools (Sguassero 2012). Energy protein supplements are used to increase the total daily protein and calorie intake in order to aid nutrition and it involves supplements in which protein provides less than 25% of the total energy content. These are available in both oral and parenteral form. Oral supplements could be in the form of whole protein milk and beverages. These supplements also contain a wide range of micronutrients which may benefit the consumer. LNS are a family of products in which majority of the energy is from lipids; they also include protein and essential fatty acids and a range of micronutrients (Dewey 2012).

### How the intervention might work

#### Nutrition education and counselling

Nutritional concerns among the adolescent age group make them vulnerable to environmental influences and consequent unhealthy eating behaviours (Riebl 2015; Stang 2017). Therefore, promotion of healthy nutrition during adolescence is vital to inculcate sustainable healthy dietary habits. Nutrition education and counselling at this stage can create knowledge through active, fun, and interactive processes and promote behaviour changes in food attitudes and practices (Baldasso 2016). Such programs can increase adolescents' ability to understand proper food practices and encourage them to actively adopt healthy food habits. It is important to note that nutrition education and counselling alone have higher chances of success if there are no other serious constraining factors in terms of access to foods and the intervention is appropriately designed for the target population group (Harrison 2010). There is some evidence that in relatively advantaged populations, targeted educational approaches can work well (Contento 1995; Harrison 2010). If provided under ideal circumstances, nutrition education and counselling have the potential to address multiple nutrient deficiencies without the risks of toxicity and interactions.

#### Micronutrient supplementation and fortification

Direct supplementation of vulnerable subpopulations with micronutrients, usually through a primary healthcare system or healthcare delivery system such as an immunization program, has been shown to be effective and cost‐effective. A direct supplementation approach through a healthcare delivery system has the advantage of directly reaching portions of the population most at risk while not putting other segments of the population at risk of overconsumption or adverse interactions (Harrison 2010). The long‐term disadvantages, however, are obvious and relate primarily to sustainability, coverage and compliance. Supplementation depends upon a viable delivery system with built‐in quality control, as well as upon wide coverage and high take‐up rates among vulnerable individuals and families. Supplementation only works if the supplements are available and accessible and the intended individuals actually take them. The risks of using dietary supplements might include organ damage from inherent toxicity, interactions, or product contamination (Harrison 2010).

The advantage to fortification of food items consumed by the general population, provided that safe and effective levels of the relevant nutrients can be delivered through an appropriate food vehicle, is that no or minimal behaviour change is required on the part of the population. This provides a tremendous advantage in terms of coverage and efficiency. Food fortification adopts an integrated approach and provides supports to improve micronutrients malnutrition when other existing food supplies fail to do so (Allen 2006).

#### Macronutrient

Supplementary feeding, balanced energy and protein supplementation and LNS are designed to increase the total daily protein and calorie intake in order to aid nutrition (Sguassero 2012). Supplementary feeding can improve the quality and quantity of the daily nutritional intake by providing additional calories, minerals and vitamins consequently leading to better nutritional status. however, there are issues of compliance, improving coverage and sustainability. It leads to the improvement in current nutritional situation and might contribute to a long term improvement but not, in and of itself, represent a solution to the primary health and nutritional problems faced by families living in poverty. Macronutrient interventions have many of the same problems as micronutrient interventions including sustainability, coverage and compliance.

We aim to assess the impact of these interventions alone or in combination on adolescent health and nutrition status in LMIC.

### Why it is important to do the review

Malnutrition is one of the most common causes of morbidity and mortality among children and adolescent population worldwide (UNICEF 2005); half of the global child mortality is attributable to malnutrition (IGME 2017). With about one quarter of the total world population (1.8 billion people) comprising adolescents and young adults (Ameratunga 2017; UNPFA 2014); it has become even more important to identify effective interventions targeting adolescents to improve their health and nutrition status to ensure sustainable healthy behaviours along with healthy growth and development (Sawyer 2012). Globally there is an increased focus on adolescents and youth as reflected by the sustainable development goals (SDGs). Existing systematic reviews assessing the impact of nutrition interventions among adolescents are either not comprehensive (assessing a single intervention or a specific micronutrient); have overlapping age groups (includes children and youth along with adolescents); or are focused on female adolescents only (Lassi 2017; Salam 2016). The majority of the existing systematic reviews have restricted their included studies to randomised trials without focusing on various contextual factors that might potentially impact the effect of nutrition interventions in this age group. Moreover, the impact of nutrition education and counselling in this age group has not been systematically reviewed. Table 1 describes the existing systematic reviews.

**Table 1:** Existing Systematic Reviews on micronutrient interventions in adolescents

**Review Article**	**Target Population**	**Intervention reviewed**	**Primary Outcomes**	**No. of studies**	**Quality Assessment**	**Meta‐Analysis MD [95% CI]**
***Salam 2016***	Adolescents (11‐19 years) and Youth (15‐24 years)	Micronutrient supplementation	Outcomes were not pre‐specified so all the outcomes reported by the study authors were included.	31	Cochrane risk of bias assessment tool	Impact of Iron‐folic acid supplementation on anaemia RR = 0.69(0.62,0.76)
***Salam 2016***	Pregnant Adolescents	Micronutrient supplementation Nutritional education	Outcomes were not pre‐specified so all the outcomes reported by the study authors were included.	16	Cochrane risk of bias assessment tool	Impact of nutrition interventions on Mean birth weight SMD = 0.25(0.08,0.41) Low birth weight RR = 0.70(0.57,0.84)
***Lassi 2017***	Adolescents (10‐19 years) and Women of reproductive age.	Micronutrient supplementation Food/protein energy supplementation Nutrition education for pregnant adolescents Obesity prevention Management of gestational diabetes	Mortality, pregnancy outcomes, morbidity, nutritional, anthropometrics	107	GRADE Working Group grades of evidence	Iron supplementation versus placebo: Haemoglobin concentration (g/L) in adolescents: SMD = 1.83(0.59,3.08) IFA supplementation versus placebo: Haemoglobin (g/L) in adolescents: MD = 2.24(0.36,4.12) Vitamin D supplementation versus placebo 25(OH)D (nmol/L) concentration in adolescents: MD = 8.80(‐2.68, 20.28) Zinc supplementation versus placebo: Haemoglobin (g/L) concentration in adolescents: SMD = 4.81(0.97,8.66) Serum zinc (mol/L) in adolescents: SMD = 4.28(2.49,6.06) Preterm birth in pregnant adolescents: RR = 0.57(0.46, 0.69) Low birth weight in pregnant adolescents: RR = 0.39(0.15,0.98) Iodine supplementation versus placebo: TSH (_U/dL) concentration in adolescents: SMD = 0.25(‐0.02, 0.52) Interventions for prevention of obesity in pregnant adolescent: birth weight: SMD = ‐0.05(‐0.11, 0.01) Interventions for management of obesity in adolescents: BMI: SMD=‐0.24(‐0.36, ‐0.13)
***Hoyland 2009***	Children or adolescent (aged 4–18 years)	Any type of breakfast manipulation	Outcome measures of cognitive performance	45	JADAD criteria used	Not performed
***Meiklejohn 2016***	Adolescents aged 10–18 years.	Nutrition education was delivered in conjunction with complementary strategies	Anthropometric measures, biochemical markers, dietary consumption data, changes in dietary intake of fruits and vegetables, snack foods, fat, sucrose, sugar‐sweetened beverages, and soft drinks.	13	American Dietetics Association. ADA Evidence Analysis Manual, IV ed.	Not performed
***Das 2013***	Children and adolescent till age of 18 years and women of reproductive age	Fortification	Serum micronutrient levels, hematologic markers, Anthropometric indicators, Pregnancy outcomes, Morbidity outcomes, Mortality	201	GRADE Working Group grades of evidence	Results for iron fortification in children haemoglobin levels: SMD = 0.55 (0.34, 0.76) Effect on anaemia: RR = 0.55 (0.42, 0.72) Results for zinc fortification in children Serum zinc levels: SMD = 1.28 (0.56, 2.01) haemoglobin level: SMD = ‐0.11(‐0.52, 0.31) Copper Levels: SMD = 0.57 (‐0.91, 2.06) Serum alkaline phosphatase levels: SMD = 0.94(‐0.29, 2.17) Weight gain: SMD = 0.50(‐0.12, 1.11) Height growth: SMD = 0.52 (0.01, 1.04) Calcium and vitamin D fortification Serum parathyroid hormone levels: SMD = ‐0.40 (‐0.56, ‐0.24) Serum vitamin D levels: SMD = 1.23 (0.35,2.11) Serum calcium levels: SMD = ‐0.40 (‐0.59, ‐0.20) Results for multiple micronutrient fortification in children haemoglobin levels: SMD = 0.75 (0.41, 1.08) Effect on anaemia: RR: 0.55 (0.42, 0.71) Effect on vitamin A deficiency: RR = 0.90 (0.76, 1.06) Height‐for age Z‐score: SMD: 0.13(‐0.04, 0.29) Weight‐for age Z‐score: SMD: ‐0.12(‐0.43, 0.20) Weight‐for height Z‐score: SMD: ‐0.11(‐0.40, 0.17) Results for iron, folate and calcium/vitamin D fortification in women haemoglobin levels: SMD: 0.62 (0.36, 0.89) Effect on anaemia: RR: 0.68 (0.49, 0.93)
***Marquez 2015***	Adolescents aged 12 to 18 years	Interventions targeting an increase in dairy food or Calcium intake	Intakes of calcium, milk and dairy per day	16	The Quality Assessment Tool for Quantitative Studies by EPHPP	Not performed
***Samuelson 2017***	Adolescents aged 10‐19 years	Diet and nutrition interventions	Depression	11	Not mentioned	Not performed
***Lohner 2012***	Children and adolescents	Folate supplementation	Serum folate content, Erythrocyte folate content	26	Not mentioned	Not performed

This review aims to comprehensively evaluate the effectiveness of all the above mentioned preventive nutrition interventions in combination or alone. We also aim to assess various contextual factors that might potentially influence the effectiveness of these nutrition interventions in this age group. This contextual information will be based on the WHO health system building blocks framework describing health systems in terms of six core components: service delivery; health workforce; health information systems; access to essential medicines/supplies; financing; and leadership/governance (WHO 2010). Findings from this review will assist the policy makers in designing contextually appropriate nutrition intervention initiatives targeting this important age group.

## Objectives

The objective of this review is to assess the impact of preventive nutrition interventions (including nutrition education and counselling, micronutrient and macronutrient supplementation) to improve the health and nutritional status of adolescents aged 10‐19 years of age in LMICs. We also aim to highlight the various contextual factors based on the WHO health system building blocks framework that might potentially impact the effectiveness of these interventions in this age group.

## Methodology

### Criteria for including and excluding studies

#### Types of study designs

We will include primary studies, including large‐scale programme evaluations, using experimental and quasi‐experimental study designs that allow for causal inference. The following study designs will be eligible for inclusion:

► Randomised controlled trials (RCTs) including both cluster and individual level randomisation
► Quasi‐experimental studies with non‐random assignment to intervention and comparison groups
► Controlled before‐after studies (CBA) studies in which observations are made before and after the implementation of an intervention, both in a group that receives the intervention and in a control group that does not.
► Interrupted time series (ITS) studies that uses observations at least three time points before and after an intervention (the ‘interruption’) to detect whether the intervention has had an effect significantly greater than any underlying trend over time.

We intend to include quasi‐experimental study designs, such as CBA and ITS, along with RCTs since we intend to assess the effectiveness of large scale program evaluations that might not be conducted in a randomised design. Moreover, we also intend to assess various contextual factors based on the WHO health system building blocks as they could potentially impact the uptake and effectiveness of these interventions.

#### Types of participants

The target population will be adolescents between 10‐19 years of age from LMICs. We will classify LMIC according to the World Bank criteria (World Bank). We will exclude studies conducted among hospitalised adolescents and adolescents with any pre‐existing health conditions. Studies including only a subset of eligible participants will be included only if the results provide information for the relevant subgroup separately.

#### Types of interventions

The following interventions alone or in any combination will be reviewed:

► Nutrition education and counselling
► Micronutrient supplementation and fortification (any micronutrient alone or in combination)
► Macronutrients supplementation

We will analyse different individual interventions separately and studies assessing a combination of interventions would be analysed separately. These will be compared with placebo/no intervention (whatever is applicable in the setting where study is conducted).

#### Types of outcome measures

We will include studies that meet our inclusion criteria, but will only include studies in the analysis that report on the following pre‐defined outcomes.

**Primary outcomes**

► Anaemia (haemoglobin less than 11 g/dL)
► Body mass index (BMI) (defined as weight in kilograms (kg) divided by height in meters squared)
► Morbidity (any morbidity as reported by the study authors for e.g. infectious diseases, night blindness etc.)
► Adverse effects. (as reported by study authors)

**Secondary outcomes**

► Serum haemoglobin levels
► Micronutrient status
► Body composition
► Development outcomes (as reported by authors; could include cognitive development, interpersonal development, and social development)
► All‐cause mortality

#### Duration of follow‐up

There will be no restrictions regarding duration of follow‐up.

#### Types of settings

Other than LMIC criteria, there will be no restrictions regarding study setting.

### Search strategy

**Electronic searches**

The search will be performed in the following electronic databases: Cochrane Controlled Trials Register (CENTRAL), MEDLINE, Embase, CINAHL, PsycINFO, the WHO nutrition databases (http://www.who.int/nutrition/databases/en/), CAB Global Health, Social Science Citation Index, Scopus, WHO Global Health Index, ADOLEC (http://bases.bireme.br/cgi‐bin/wxislind.exe/iah/adolec/?IsisScript=iah/iah.xis&base=ADOLEC&lang=i&form=A), EPPI (http://bases.bireme.br/cgi‐bin/wxislind.exe/iah/adolec/?IsisScript=iah/iah.xis&base=ADOLEC&lang=i&form=A). The trials registry Clinicaltrials.gov will be searched for ongoing trials. We will search Google Scholar along with key nutrition agencies database such as Nutrition International, the Global Alliance for Improved Nutrition, the World Food Programme, and HarvestPlus to search for non‐indexed, grey literature to locate relevant programme evaluations and any additional trials. We will not apply any restrictions based on publication date, language or publication status.

**Searching other resources**

We will make every effort to contact relevant organisations and experts in the field to identify unpublished or ongoing studies. We will also search Eldis.org to find organisations with an interest in nutrition. References of included articles, relevant reviews, and annotated bibliographies will be scanned for eligible studies. We will run citation searches of included studies in Google Scholar to identify any recent studies missed from the database searches.

### Description of methods used in primary research

For this review, we will include primary studies assessing the effectiveness of aforementioned nutrition interventions among adolescents aged 10‐19 years of age. The study designs of interest include RCTs as well as non‐randomised studies including quasi experimental studies, CBA studies and ITS studies. For example, the study by Ziauddin Hyder 2007 is a potentially eligible study. This study is a randomised, double‐blind, placebo‐controlled trial assessing the effectiveness of a multiple‐micronutrient‐fortified beverage. The participants in this study were 1125 adolescent girls from 54 non‐formal primary education schools in rural Bangladesh. The outcomes of interest included haemoglobin concentrations, micronutrient status, and growth among adolescent girls.

### Criteria for determination of independent findings

Before the initiating the synthesis (detailed below), we will ensure that all articles reporting on the same study are appropriately linked. To ensure independence and appropriate combination of outcome constructs, syntheses will be conducted according to the type of interventions specified above. If multi‐arm studies are included, intervention groups will be combined or separated into different forest plots, and we will ensure that there is no double counting of participants. If an outcome is reported in several different metrics, we will perform unit conversions in order to pool the data. We do anticipate differences in the types of literature and we will ensure that any analysis will take possible sources of dependency into account by grouping papers into studies and ensuring that no double counting of evidence takes place when synthesizing across studies.

### Details of study coding categories

Two review authors (RAS and OI) will extract data independently and a third review author (JKD) will check for reliability and resolve any conflict. We will extract the primary data for the study characteristics including details of the populations, setting, socio‐demographic characteristics, interventions, comparators, outcomes and study design in duplicate. We will check primary study data for accuracy. Disagreements will be resolved by discussion or consultation with a third reviewer.

The following information will be extracted for each included study:

► Background: time period when study took place, type of publication (e.g. full‐text journal article, abstract, conference paper, thesis), study country or countries, funding source(s), and conflicts of interest
► Population and setting: population age and setting
► Methods: Study design, description of study arms, unit of allocation, sample or cluster size per study arm (for individually or cluster randomised trials respectively), start and end date, follow‐up
► Participants: total number randomised/allocated, sample representativeness, baseline characteristics, number of withdrawals, socio‐demographic data
► Intervention group details: number randomised/allocated to group, description of intervention, duration and follow‐up, timing, delivery of intervention, providers and their training. We will describe all the study intervention arms in the tables of included studies, however, we will only report the intervention arms that meet review inclusion criteria.
► Comparison group details: number randomised to group, description of comparison, duration and follow‐up, timing, providers and their training
► Outcomes: measurement tool, validation of the tool, total number in intervention and comparison groups, change indicated at each time point
► Other information

In addition to the above mentioned details, we will also collect details related to the program related contextual factors. This information will be based on the WHO health system building blocks framework describing health systems in terms of six core components (WHO 2010):

► Service delivery: The availability of health services including all services dealing with the delivery of nutrition interventions.
► Health workforce: The availability of sufficient and capable staff to deliver nutrition interventions.
► Health information systems: The availability of the production, analysis, dissemination and use of reliable and timely information on health and nutrition related determinants and status.
► Access to essential medicines/supplies: The availability of nutrition intervention related commodities and supplies in adequate amounts, in the appropriate dosages and at an affordable price.
► Financing: The sources of funds available for the delivery of nutrition interventions.
► Leadership/governance: The roles and responsibilities of various sectors including public, private and voluntary sectors in implementing the nutrition interventions.

### Assessment of risk of bias in included studies

For RCTs we will use the Cochrane risk of bias tool (Higgins 2011) which assesses selection bias, performance bias, detection bias, attrition bias and reporting bias. We will rate each component as ‘high’, ‘low’, or ‘unclear’ for each risk of bias component. For non‐randomised studies, we will use the Cochrane Effective Practice and Organisation of Care (EPOC) risk of bias criteria (based on additional criteria including similar baseline outcome measurements, similar baseline characteristics, knowledge of the allocated interventions adequately prevented during the study, protection against contamination, intervention independent of other changes, shape of intervention effect pre‐specified and intervention unlikely to affect data collection) and rate the studies as low risk, high risk or unclear risk (EPOC 2017). We will provide supporting evidence for the risk of bias judgements. Two independent reviewers will perform quality appraisal for each study and disagreements will be resolved by discussion or consultation with a third reviewer. We will summarise the quality of evidence according to the outcomes as per the Grading of Recommendations, Assessment, Development and Evaluation (GRADE) criteria (Walker 2010). A grade of ‘high’, ‘moderate’, ‘low’ and ‘very low’ will be used for grading the overall evidence indicating the strength of an effect on specific health outcome based on methodological flaws within the component studies, consistency of results across different studies, generalizability of research results to the wider patient base and how effective the treatments have shown to be (Balshem 2011). For non‐randomised studies, the evidence quality will be updated [upgraded?] based on large magnitude of effect, dose response [dose‐response relationship?] and effect of all plausible confounding factors would be to reduce the effect (where an effect is observed) or suggest a spurious effect (when no effect is observed). Two reviewers will discuss ratings and reach consensus, and disagreements will be resolved by consulting a third reviewer. We will develop a summary of findings table to show the effects for the primary outcomes.

### Statistical procedures and conventions

Following synthesis procedures and analysis methods will be used:

**Measures of treatment effect**

We will perform statistical analysis using RevMan 5 (Revman 2014). For dichotomous data, we will use odds ratios (OR), and risk ratios (RR) with 95% confidence intervals (CI). For continuous data, we will use the mean difference (MD) with 95% CI, if outcomes are measured in the same way between trials. We will use the standardized mean difference (SMD) with 95% CI to combine trials that measure the same outcome but use different methods of measurement.

**Unit of analysis issues**

In case if the trials have reported the outcomes of interest at multiple time points, we will report the outcome from the last time point. Where trials have used clustered randomisation, we anticipate that study investigators would have presented their results after appropriately controlling for clustering effects (for example, variance inflated standard errors, hierarchical linear models). If it is unclear whether a cluster‐ randomised controlled trial has appropriately accounted for clustering, the study investigators will be contacted for further information. Where appropriate controls for clustering were not used, we will request an estimate of the intra‐class correlation coefficient. The data will be re‐analysed using multi‐level models which control for clustering. Following this, effect sizes and standard errors will be meta‐analysed in RevMan using the generic inverse method (Higgins 2011a). They will be combined with estimates from individual level trials. We will use sensitivity analyses to assess the potential biasing effects of using the interclass correlation coefficients that have been derived in different ways.

**Dealing with missing data**

If the outcome of interest does not include data on all participants, we will first contact the study authors via email to inquire about data for the missing cases. Missing data, if found, will be re‐included in the analysis. If unable to find missing data we will analyse data for only those participants whose results are available, and address the impact of the missing data in the assessment of risk of bias.

**Assessment of heterogeneity**

We will assess heterogeneity among studies in two ways. Firstly, we will assess heterogeneity at face value: heterogeneity in population, interventions, or outcomes. We will use I^2^, Q, and tau^2^ statistics as a guide to assess heterogeneity along with a visual inspection of forest plots.

**Assessment of reporting biases**

Funnel plots would be used if there are 10 or more studies in meta‐analysis for one outcome, investigation will be conducted for reporting biases for example publication bias.

**Data synthesis**

A meta‐analysis will be conducted separately for each outcome and intervention. Furthermore, for each outcome, we will separately meta‐analyse for different study designs (RCT, ITS and CBA). We will pool data from studies we judge to be clinically homogeneous, if more than one study provides usable data in any single comparison, we will perform a meta‐analysis. We will standardize all the reported effect sizes as RRs for the dichotomous outcome and SMDs for the continuous outcomes. We will attempt to standardise the outcomes as a common metric and synthesize together where possible. We will carry out statistical analysis using the Review Manager software (Revman 2014). We will use random‐effects meta‐analysis for combining data to produce an overall summary, since we expect reasonable clinical heterogeneity in interventions, comparisons, outcomes, or settings within the studies included. The random‐effects summary will be treated as the average of the range of possible treatment effects and we will discuss the clinical implications of treatment effects differing between trials. If the average treatment effect is not clinically meaningful, we will not combine trials. We will report statistical heterogeneity as I^2^, Q, tau^2^ statistics for all random‐effects meta‐analyses. We will narratively synthesize and report the findings from the contextual factors based on the WHO health system building blocks framework for each intervention.

### Subgroup analysis and investigation of heterogeneity

Based on the availability of the data, subgroup analysis will be conducted for following subgroups:

► Duration or intensity of intervention (e.g. short versus long term, one‐off versus multiple sessions).
► Individual context versus group context (for nutrition education and counselling only i.e. children receive the intervention individually versus those in groups)
► Study setting: school, community, clinic etc.
► Sex: Male and females.
► Population (e.g. urban population versus rural population; resource poor versus resource rich population)
► We will also attempt to conduct subgroup analysis based on the WHO health system building blocks factors (where data is available).

We will assess difference in subgroups based on the methodology described in the Cochrane Handbook (Higgins 2011) by using a simple approach for a significance test to investigate differences between two or more subgroups. We will undertake a standard test for heterogeneity across subgroup results using Chi^2^ test or moderator analysis rather than across individual study results.

### Sensitivity analysis

Sensitivity analyses will be performed to consider the impact of the following:

► Allocation concealment (adequate versus inadequate and/or unclear).
► Attrition (< 20% versus >= 20%).

### Treatment of qualitative research

We do not plan to include qualitative research.

## Review authors

**Lead review author:**

**Table:** 

Name:	Rehana A Salam
Title:	Ms.
Affiliation:	South Australian Health and Medical Research Institute, University of Adelaide and Aga Khan University
Address:	University of Adelaide, Australia
City, State, Province or County:	Adelaide
Post code:	5000
Country:	Australia
Phone:	451321440
Email:	rehana.salam@aku.edu
**Co‐authors:**	
Name:	Jai K Das
Title:	Dr
Affiliation:	Aga Khan University
Address:	Stadium Road
City, State, Province or County:	Karachi
Post code:	74800
Country:	Pakistan
Phone:	21‐34930051
Email:	jai.das@aku.edu
	
Name:	Omar Irfan
Title:	Dr
Affiliation:	Aga Khan University
Address:	Stadium Road
City, State, Province or County:	Karachi
Post code:	74800
Country:	Pakistan
Phone:	????
Email:	omarirfan1@hotmail.com
	
Name:	Zulfiqar A Bhutta
Title:	Dr
Affiliation:	Centre for Global Child Health, The Hospital for Sick Children, Toronto, Canada and Centre of Excellence in Women and Child Health, Aga Khan University, Karachi, Pakistan.
Address:	SickKids
City, State, Province or County:	Toronto
Post code:	M5G 1X8
Country:	Canada
Phone:	416‐813‐7654 ext. 328532
Email:	zulfiqar.bhutta@sickkids.ca

## Roles and responsibilities

Please give a brief description of content and methodological expertise within the review team. It is recommended to have at least one person on the review team who has content expertise, at least one person who has methodological expertise and at least one person who has statistical expertise. It is also recommended to have one person with information retrieval expertise. Please note that this is the *recommended optimal* review team composition.

► Content: Rehana A Salam, Jai K Das, Zulfiqar A Bhutta
► Systematic review methods: Rehana A Salam, Jai K Das
► Statistical analysis: Rehana A Salam, Jai K Das
► Information retrieval: Rehana A Salam, Omar Irfan

## Sources of support

Funding for this review came from a grant from the Bill & Melinda Gates Foundation to the Centre for Global Child Health at The Hospital for Sick Children (Grant No. OPP1137750).

## Declarations of interest

Please declare any potential conflicts of interest. For example, have any of the authors been involved in the development of relevant interventions, primary research, or prior published reviews on the topic?

None to declare.

## Preliminary timeframe

Approximate date for submission of the systematic review. January 20, 2019

Please note this should be no longer than two years after protocol approval. If the review is not submitted by then, the review area may be opened up for other authors.

## Plans for updating the review

Reviews should include in the protocol specifications for how the review, once completed, will be updated. This should include, at a minimum, information on who will be responsible and the frequency with which updates can be expected.

Rehana A Salam will be responsible for updating this review and the review will be update every two years after publication date.

## AUTHOR DECLARATION

### Authors' responsibilities

By completing this form, you accept responsibility for preparing, maintaining and updating the review in accordance with Campbell Collaboration policy. Campbell will provide as much support as possible to assist with the preparation of the review.

A draft review must be submitted to the relevant Coordinating Group within two years of protocol publication. If drafts are not submitted before the agreed deadlines, or if we are unable to contact you for an extended period, the relevant Coordinating Group has the right to de‐register the title or transfer the title to alternative authors. The Coordinating Group also has the right to de‐register or transfer the title if it does not meet the standards of the Coordinating Group and/or Campbell.

You accept responsibility for maintaining the review in light of new evidence, comments and criticisms, and other developments, and updating the review at least once every five years, or, if requested, transferring responsibility for maintaining the review to others as agreed with the Coordinating Group.

### Publication in the Campbell Library

The support of the Coordinating Group in preparing your review is conditional upon your agreement to publish the protocol, finished review, and subsequent updates in the Campbell Library. Campbell places no restrictions on publication of the findings of a Campbell systematic review in a more abbreviated form as a journal article either before or after the publication of the monograph version in Campbell Systematic Reviews. Some journals, however, have restrictions that preclude publication of findings that have been, or will be, reported elsewhere and authors considering publication in such a journal should be aware of possible conflict with publication of the monograph version in Campbell Systematic Reviews. Publication in a journal after publication or in press status in Campbell Systematic Reviews should acknowledge the Campbell version and include a citation to it. Note that systematic reviews published in Campbell Systematic Reviews and co‐registered with Cochrane may have additional requirements or restrictions for co‐publication. Review authors accept responsibility for meeting any co‐publication requirements.

**I understand the commitment required to undertake a Campbell review, and agree to publish in the Campbell Library. Signed on behalf of the authors**:

**Table:** 

**Form completed by: Rehana A Salam**	**Date: 17 October 2018**

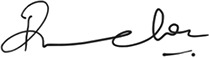

## Appendix

***1 Search Strategy***

**PubMed Search Strategy:** (titles/abstracts and text words)

((“Adolescent”[Mesh]) OR (“Child”[Mesh]) OR (Adolescent* OR Adolescence) OR (Teen* OR (Youth*) OR (Puberty) OR (juvenil*)) AND ((“Micronutrients”[Mesh]) OR (“Dietary Supplements”[Mesh]) OR (“Food, Fortified”[Mesh]) OR (“Vitamins”[Mesh]) OR (“Minerals”[Mesh] OR “Trace Elements”[Mesh]) OR (“Ferric compounds”[Mesh] OR “Ferrous Compounds”[Mesh]) OR (Iron* OR Ferric OR Ferrous) OR (“Diet Supplement*” OR “Dietary Supplement*” OR Biofortification) OR (“Folic Acid”[Mesh]) OR (Folic* OR Folate* OR Folvite* OR Folacin*) OR (“Zinc”[Mesh] OR “Zinc Sulfate”[Mesh]) OR (“Calcium”[Mesh]) OR (Calcium) OR (“Vitamin D”[Mesh]) OR (vitamin d) OR (“Vitamin A”[Mesh]) OR (“Vitamin A”) OR (“Ascorbic Acid”[Mesh]) OR (“Vitamin C”) OR (Ascorb* OR “ascorbic acid”) OR (Vitamin* OR multivitamin* OR multi‐vitamin* OR MMN OR micro‐nutrient* OR mineral* OR multimineral* OR multi‐mineral OR multinutrient* OR “multiple micronutrient*” OR “food environment” OR advertisement* OR “mass media” OR “supplementary feeding” OR “energy supplement*” OR “protein supplement*” OR “lipid based nutrition” OR LNS)) AND ((“Adolescent Development”[Mesh]) OR (“Adolescent Growth”) OR (“Serum Haemoglobin” OR “Serum micronutrient*” OR “Anthropometric measurement*”))

**EBSCO CINAHL Plus:**

((“Adolescent”[Mesh]) OR (Adolescent* OR Adolescence) OR (Teen* OR Teenager*) OR (Youth*) OR (Puberty) OR (juvenile)) AND ((“Micronutrients”[Mesh]) OR (“Dietary Supplements”[Mesh]) OR (“Food, Fortified”[Mesh]) OR (“Vitamins”[Mesh]) OR (“Minerals”[Mesh] OR “Trace Elements”[Mesh]) OR (“Ferric compounds”[Mesh] OR “Ferrous Compounds”[Mesh]) OR (Iron* OR Ferric OR Ferrous) OR (“Diet Supplement*” OR “Dietary Supplement*” OR Biofortification) OR (“Folic Acid”[Mesh]) OR (Folic* OR Folate* OR Folvite* OR Folacin*) OR (“Zinc”[Mesh] OR “Zinc Sulfate”[Mesh]) OR (“Calcium”[Mesh]) OR (Calcium) OR (“Vitamin D”[Mesh]) OR (vitamin d) OR (“Vitamin A”[Mesh]) OR (“Vitamin A”) OR (“Ascorbic Acid”[Mesh]) OR (“Vitamin C”) OR (Ascorb* OR “ascorbic acid”) OR (Vitamin* OR multivitamin* OR multi‐vitamin* OR MMN OR micro‐nutrient* OR mineral* OR multimineral* OR multi‐mineral OR multinutrient* OR “multiple micronutrient*” OR “food environment” OR advertisement* OR “mass media” OR “supplementary feeding” OR “energy supplement*” OR “protein supplement*” OR “lipid based nutrition” OR LNS)) AND ((“Adolescent Development”[Mesh]) OR (“Adolescent Growth”) OR (“Serum Haemoglobin” OR “Serum micronutrient*” OR “Anthropometric measurement*”))

**Cochrane Library**:

((“Adolescent”[Mesh]) OR (“Child”[Mesh]) OR (Adolescent* OR Adolescence) OR (Teen* OR (Youth*) OR (Puberty) OR (juvenil*)) AND ((“Micronutrients”[Mesh]) OR (“Dietary Supplements”[Mesh]) OR (“Food, Fortified”[Mesh]) OR (“Vitamins”[Mesh]) OR (“Minerals”[Mesh] OR “Trace Elements”[Mesh]) OR (“Ferric compounds”[Mesh] OR “Ferrous Compounds”[Mesh]) OR (Iron* OR Ferric OR Ferrous) OR (“Diet Supplement*” OR “Dietary Supplement*” OR Biofortification) OR (“Folic Acid”[Mesh]) OR (Folic* OR Folate* OR Folvite* OR Folacin*) OR (“Zinc”[Mesh] OR “Zinc Sulfate”[Mesh]) OR (“Calcium”[Mesh]) OR (Calcium) OR (“Vitamin D”[Mesh]) OR (vitamin d) OR (“Vitamin A”[Mesh]) OR (“Vitamin A”) OR (“Ascorbic Acid”[Mesh]) OR (“Vitamin C”) OR (Ascorb* OR “ascorbic acid”) OR (Vitamin* OR multivitamin* OR multi‐vitamin* OR MMN OR micro‐nutrient* OR mineral* OR multimineral* OR multi‐mineral OR multinutrient* OR “multiple micronutrient*” OR “food environment” OR advertisement* OR “mass media” OR “supplementary feeding” OR “energy supplement*” OR “protein supplement*” OR “lipid based nutrition” OR LNS)) AND ((“Adolescent Development”[Mesh]) OR (“Adolescent Growth”) OR (“Serum Haemoglobin” OR “Serum micronutrient*” OR “Anthropometric measurement*”))
